# Elucidation of the mechanism of partial activation of EPAC1 allosteric modulators by Markov state modelling†

**DOI:** 10.1039/d5sc02112j

**Published:** 2025-07-04

**Authors:** Adele Hardie, Frederick G. Powell, Silvia Lovera, Stephen J. Yarwood, Graeme Barker, Julien Michel

**Affiliations:** a EaStCHEM School of Chemistry, University of Edinburgh David Brewster Road Edinburgh EH9 3FJ UK julien.michel@ed.ac.uk; b Institute of Chemical Sciences, Heriot-Watt University Riccarton Edinburgh EH14 4AS UK; c UCB Chemin du Foriest 1, Braine-l'Alleud 1420 Belgium; d Institute of Biological Chemistry, Biophysics, and Bioengineering, Heriot-Watt University Riccarton Edinburgh EH14 4AS UK

## Abstract

The development of selective modulators of exchange protein activated by cAMP (EPAC1/RAPGEF3) would pave the way for novel therapeutic interventions in cardiac, metabolic, inflammatory, and oncologic disorders. Here we have applied a computational workflow using Markov State Models (MSMs) and steered molecular dynamics (sMD) to probe the allosteric activation of EPAC1 by both cAMP and pharmaceutical hit compound I942. sMD was used to examine the large-scale domain rearrangement EPAC1 undergoes during activation. Intermediate conformations accessed *via* sMD were then used as starting points for equilibrium MD simulations, which were pooled for the construction of MSMs. The resulting models capture the activation of wild-type (WT) EPAC1 by cAMP, and provide an explanation for the lack of response to cAMP shown by the L273W point mutant. sMD/MSM modelling also elucidated the structural basis for partial activation of EPAC1 by ligand I942 and revealed the crucial contribution of ligand interactions with EPAC1's catalytic region to achieve full activation. The mechanistic insights from this study suggest a design strategy to guide the development of potent small-molecule EPAC1 activators.

## Abstract 2

The development of selective modulators of exchange protein activated by cAMP (EPAC1/RAPGEF3) would pave the way for novel therapeutic interventions in cardiac, metabolic, inflammatory, and oncologic disorders.

## Graphical abstract

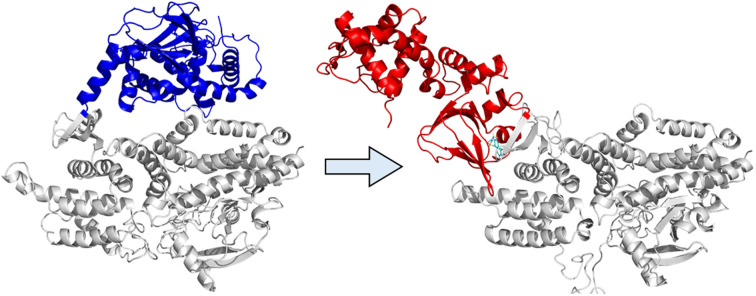

## Introduction

EPAC1 (RAPGEF3) is a guanine nucleotide exchange factor for the small GTPases Rap1 and Rap2 that is directly activated by cAMP, offering a broader scope of cAMP effects beyond the canonical PKA pathway.^1,2^ Recent work underscores EPAC1's diverse roles in cell–cell adhesion, endothelial barrier function, immune cell trafficking, and metabolic pathways,^3^ including a striking demonstration that it enhances brown fat growth and beige adipogenesis.^3^ In cardiomyocytes, EPAC1-mediated regulation of intracellular calcium handling influences both contractile performance and arrhythmic risk, suggesting therapeutic relevance in heart failure.^4^ Moreover, EPAC1 activation in endothelial cells strengthens adherens junctions through Rap1 signalling, thereby mitigating vascular leakage under inflammatory or ischaemic conditions.^5^ In pancreatic β-cells, EPAC1 contributes to insulin secretion and cytoprotective signalling, offering a potential target for managing type 2 diabetes.^6^ In oncology, its effect is more context-dependent, with EPAC1 activation reinforcing normal adherens junctions in some cases yet facilitating tumour invasion in others, as shown by the suppression of metastatic spread upon EPAC1 inhibition in pancreatic cancer models.^7^ The development of highly selective EPAC1 modulators therefore paves the way for novel therapeutic interventions.^8^

**Fig. 1:**
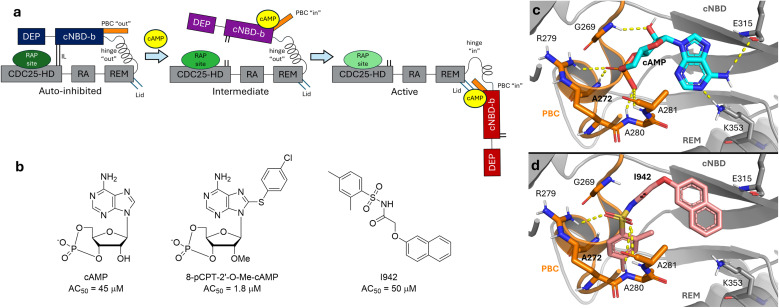
Structure of inactive, intermediate, and active EPAC1, and activators. (a) *apo* EPAC1 exists in an auto-inhibited state, with the RR (blue) blocking the RAP binding site (dark green) on the CR (gray). Upon binding of cAMP, the PBC shifts from “out” to “in” and the RR begins to move away (purple). The hinge can then open, fully relocating the RR (red) and exposing the RAP binding site (light green) once the hinge is fully open, cAMP engages in interactions with the “lid” region, on the REM immediately C-terminal to the hinge. (b) Activators of EPAC1: cAMP, 8-pCPT-2′-*O*-Me-cAMP (EPAC1-selective cAMP analogue), and I942, the first non-cAMP analogue partial activator. (c) The modelled binding pose of cAMP (cyan) to EPAC1 in the active conformation (orange), based on structure of cAMP analogue Sp-cAMP with EPAC2 (PDB ID: 3CF6). Key interactions are shown as yellow dashes. (d) The modelled binding pose of I942 (pale green) to EPAC1 in the active conformation (orange). Key interactions are shown as yellow dashes.

EPAC1 is comprised of a regulatory region (RR), containing the allosteric cAMP binding site, and a catalytic region (CR), containing the Rap binding (active) site.^9^ The CR is comprised of the RAS-exchange motif (REM), the RAS association (RA), and CDC25 homology domain (CDC25HD). When Rap1 binds to EPAC1, the nucleotide binding site on Rap1 becomes deformed, decreasing both GDP and GTP affinity. The significantly higher GTP concentration in the cell favours GTP binding over GDP.^10^ The RR of EPAC1 contains Disheveled Egl-10 Plecstrin (DEP) and a single cyclic nucleotide binding domain (cNBD) (Fig. 1a). In the absence of cAMP, EPACs exist in an auto-inhibited state, where the RR is blocking the Rap binding site on the CR. The hinge between the two regions adopts a helical conformation, while the adjacent phosphate binding cassette (PBC) sterically blocks the hinge from moving and revealing the Rap binding site.^11^ The coupling of the conformational changes between the PBC and hinge is highlighted in the L273W mutant. Replacement of the L273 residue on the PBC with a bulky tryptophan mutation prevents the hinge adopting the active conformation, and no guanine nucleotide exchange factor activity was observed in EPAC1_L273W_, even in the presence of 500 μM cAMP.^12^ The interface between the RR and the CR is also stabilized *via* a mixture of hydrogen bonding and ionic interactions between residues of the two domains (ESI Fig. 1†) (ionic latch, or IL), and the helical conformation of the hinge prevents opening of EPAC1.^9,13^ Upon binding of cAMP, the PBC shifts from an “out” to an “in” conformation (Fig. 1a). This allows the hinge helix to unfold at its C-terminus end, moving the RR by approximately 45 Å and enabling RAP binding to the exposed active site (Fig. 1a). Additionally, the active conformation of EPAC1 is further stabilized by the interactions between the adenosine group of cAMP and K353 of the REM domain on the catalytic region, as well as E315 of the C-terminus of the cNBD (Fig. 1c). The terminal β-sheet strands of the cNBD and the first helix of the REM domain are known as the “lid”, as they close off the previously solvent-exposed cAMP binding site upon activation.^11^

To summarise, the two main structural features that discriminate between EPAC1 inactive and active conformations (Fig. 1a) are: the PBC region (“out” to “in” transition), and the hinge region (“out” to “in” transition).

Initial efforts to drug EPAC1 were focused on design of cAMP analogues,^14–19^ such as 8-pCPT-2′-*O*-Me-cAMP, an EPAC1-selective agonist^18,20^ (Fig. 1b). However, these suffered from poor membrane permeability due to the presence of a negatively-charged phosphate group. To overcome this liability, the negative charge was masked with an acetoxymethyl ester, which is hydrolysed by cytosolic esterase enzymes to return the active agent.^17^ Whilst this improved bioavailability, some cAMP analogues were also associated with off-target effects,^21^ particularly non-selective activation of EPAC2 and PKA. These side effects have shifted the focus of activating EPAC1 to non-cAMP-like small molecules,^22^ leading to the development of I942 (Fig. 1b and d), a selective partial EPAC1 agonist.^23^ I942 was identified in a high-throughput screen (HTS) of lead-like small molecules, using a competitive displacement assay of fluorescent cAMP analogue 8-NBD-cAMP. I942 induces partial activation of EPAC1 in cells.^24^ I942 is also more drug-like compared to cAMP analogues, and provides a more suitable scaffold for further lead optimisation towards a full selective activator. NMR studies have suggested that while I942 binds to the same site as cAMP, the partial activation of EPAC1 arises from the stabilisation of an intermediate state, rather than the active conformation of EPAC1. In this intermediate state, the PBC has adopted an “in”, or active-like, conformation, but the hinge in the cNBD has not yet shifted and remains in the “out”, or inactive-like” conformation.^25^

While the NMR work done on EPAC1–I942 has provided evidence for the intermediate state stabilisation to explain the partial activation observed, the data on I942 is still sparse and limited by the experimental techniques employed. Due to poor stability of full length EPAC1, only the more stable isolated EPAC1–cNBD was used for the original HTS screen that identified I942.^23^ When the full length protein was used in the activation assay, cAMP affinity was found to be 10-fold higher than seen previously with just the cNBD, whereas I942 affinity to both structures was very similar.^23^ Additionally, the work by Shao *et al.* also used only EPAC1–cNBD (residues 149–318), which precluded assessment of the significance of ligand interactions with the catalytic region (such as K353 shown in Fig. 1c and d).

This report presents molecular dynamics (MD) simulations to fill the gaps in available experimental data and provide atomistic resolution insight into the conformational dynamics of EPAC1 activation. Markov State Modelling (MSM) is a powerful approach to construct atomistic models of the energetics and kinetics of biomolecular systems.^26–33^ In previous work, we have combined enhanced sampling MD with MSM to account for changes in protein conformational ensembles in the presence of allosteric inhibitors.^34^ However, this preceding work only covered small loop motions, rather than large domain rearrangements that characterise EPAC activity. Here we use steered molecular dynamics (sMD) to simulate the conformational change of EPAC1 from inactive to active states (and *vice versa*), *via* the intermediate state previously identified by Shao *et al.*^25^ These calculations were used to initiate an ensemble of unbiased MD simulations, starting from various points along the sMD coordinate. This unbiased MD dataset is subsequently used to build MSMs and coarsen the description of the conformational change to a three macrostates (“inactive”, “intermediate”, “active”) partitioning of the EPAC1 conformational ensemble. As this methodology has only been applied to inhibitors previously, it is validated by first comparing *apo* WT EPAC1 to the EPAC1–cAMP complex, as well as EPAC1_L273W_–cAMP. Our modelling correctly captures the activation of WT EPAC1 by cAMP, and also the prevention of the activation by the L273W mutation. Following this, we investigate the effects of I942 on EPAC1 by modelling I942 with native interactions only, as well as with additional artificial protein–ligand distance restraints to mimic hydrogen bonding interactions observed with cAMP but not I942. Comparisons of the computed conformational ensembles allowed us to isolate and determine the effects of key cAMP–EPAC1 interactions. Finally, we propose structural modifications that could convert I942 derivatives into full EPAC1 agonists.

## Methods

**Fig. 2:**
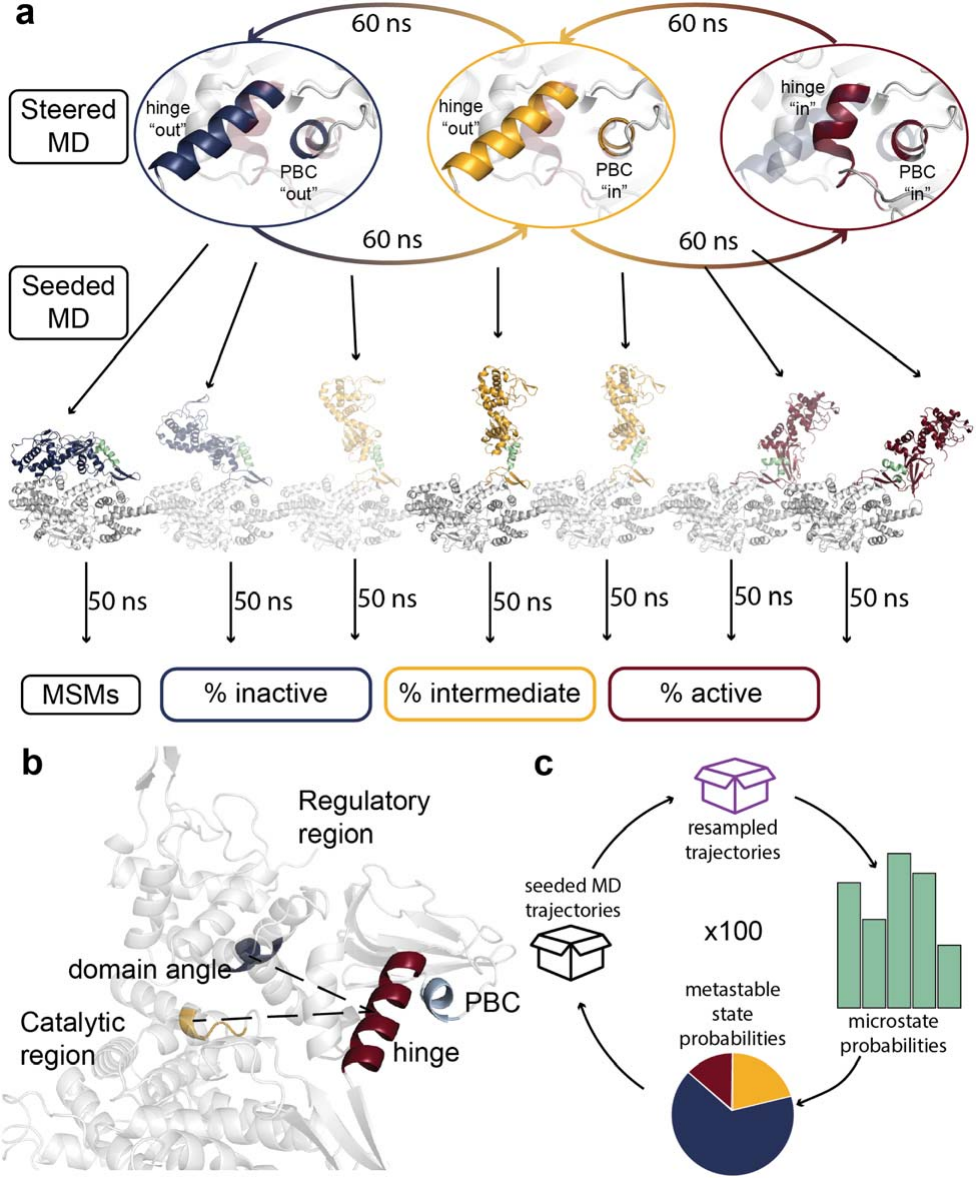
The sMD/MSM workflow applied to EPAC1. (a) sMD was used to drive EPAC1 from inactive to active state and *vice versa*. In the examples shown in this figure, the RR is highlighted (hinge and PBC shown in green), while the CR is coloured in grey. Further unbiased 50 ns MD simulations (seeded MD) were run using each of the conformation snapshots as starting coordinates, which were used to build the MSMs. (b) The features used to reduce MD data dimensionality. cNBD–hinge–CR domain angle (shown in dashed black lines), hinge (red) RMSD and PBC (light green) RMSD, both to inactive conformation. (c) The bootstrapping cycle. At each iteration the seeded MD pool of trajectories for each system is randomly resampled with replacement to select 200 trajectories.

The overall workflow for the sMD/MSM methodology used in this study is depicted in Fig. 2.

### Protein modelling

The homology models of EPAC1 in the active and inactive conformations were prepared using the SWISS-MODEL webserver.^35^ The FASTA sequence was obtained from the UniProt database (entry O95398). X-ray diffracted structures of EPAC2 were used as templates when constructing the protein models: EPAC2–cAMP analogue complex (PDB ID: 4MGK) and *apo* EPAC2 (PDB ID: 2BYV) for active and inactive conformations respectively. The active conformation template lacked the DEP domain, which therefore was copied from the inactive conformation using PyMol. All protein structures were capped with ACE and NME caps for the N- and C-termini respectively using the software Flare.^36^ In all cases histidines 439 and 646 (model residues 392 and 599) were modelled as protonated at the *δ* position based on propka3 (ref. 37) predicted p*K*_a_ values. The EPAC1 model in its inactive conformation was compared to an AlphaFold generated structure. With the exception of large disordered loop regions present in the AlphaFold model, the two structures were found to be in high agreement (ESI Fig. 2†). The DEP-CNBD of both active and inactive models also aligned well (backbone RMSD 3.3 Å and 3.4 Å respectively) with a separate crystal structure of the EPAC1 regulatory region (PDB ID:6H7E). RMSD values were computed using cpptraj v4.25.6 as distributed in AmberTools22.^38^

### Ligand modelling

Cyclic AMP was manually docked to the active conformation of EPAC1 by aligning the EPAC2 complex with the cAMP analogue (Sp-cAMP) in the PDB entry 3CF6, and editing it with the software Flare.^36^ cAMP was further manually docked to the inactive conformation of EPAC1 by aligning the cNBD regions of the protein using PyMol and copying the ligand coordinates. Compound I942 was manually docked *in situ* by editing the previously obtained cAMP coordinates in Flare. The ligand pose was in agreement with NMR measurements from Shao *et al.* that indicated formation of hydrogen bonds between I942 and EPAC1 residues R279, A280 and A281.^25^

### System preparation

All system setup was carried out *via* BioSimSpace.^39^ The proteins were parameterized using the AMBER ff14SB forcefield, and GAFF2 with the AM1-BCC charge method was used for the ligands. cAMP was modelled with a charge of −1, and I942 was modelled as neutral. All systems were solvated in TIP3P water with a 15 Å shell and a 150 mM NaCl concentration, adding ions as needed to neutralize the system. In all cases, energy minimization was carried out for 7500 steps, heating to 300 K for 500 ps, and further equilibration for 2 ns, all using GROMACS 2020.2.^40^ Particle Mesh Ewald (PME) was used, with a direct space cutoff of 12 Å.

### Equilibrium molecular dynamics

Simulations of *apo* EPAC1 in active and inactive conformations were carried out for 1 μs each, using pmemd.cuda from AMBER22.^41^ Trajectories were written out every 10 ps, and simulations were run at 300 K temperature and 1 atm pressure, using Langevin dynamics with *γ* = 2 ps^−1^. Features later used for MSM building (section) were computed using cpptraj.

### Steered molecular dynamics

Steered molecular dynamics simulations^42^ were performed using pmemd.cuda from AMBER22 (ref. 41) with PLUMED v2.6.1,^43,44^ using BioSimSpace for input file preparation. Tutorials to implement sMD simulations with BioSimSpace are available elsewhere.^45^ Simulations were run at a temperature of 300 K and 1 atm pressure, using Langevin dynamics with *γ* = 2 ps^−1^. Steering was carried out in both directions in two steps, *i.e.* inactive–intermediate–active and active–intermediate–inactive (Fig. 1b). The steering CVs were regulatory region backbone RMSD to the final target conformation (active or inactive), hinge backbone RMSD to step target conformation (active, intermediate, or inactive), and PBC backbone RMSD to step target conformation (active, intermediate, or inactive). The reference for hinge and PBC in all cases included the cNBD up to and including the hinge region (residues 122–263 of the protein model). The reference for the intermediate state was prepared using PyMol by replacing the PBC (residues 270–274, model residues 223–227) of the inactive conformation with the one from the active conformation. No additional energy minimisation was required. The force constant applied to all CVs was 3500 kJ mol^−1^, and the simulation duration was 60 ns for each step (120 ns total). sMD parameters are shown in ESI Table 1.†

During preliminary runs, spontaneous dissociation of both cAMP and I942 was observed. Therefore, to maintain relevant binding poses and prevent ligand dissociation during the movement of the cNBD region, both cAMP and I942 were restrained using flat bottomed restraints during all sMD simulations. The ligand restraints are shown in ESI Table 2.†

### Seeded molecular dynamics

The two steering trajectories were combined into a single trajectory and 100 snapshots were extracted from each steering direction, equally sampling the CVs used for steering, using cpptraj. These conformations were used as starting points (or “seeds”) for a further 50 ns of equilibrium MD simulations with pmemd.cuda (AMBER22). Trajectories were written out every 10 ps, and simulations were run at 300 K temperature and 1 atm pressure, using Langevin dynamics with *γ* = 2 ps^−1^. For the I942 restrained systems, some ligand restraints were also maintained during the seeded MD simulations to mimic the effects of additional protein–ligand interactions (ESI Table 2 and ESI Fig. 3†).

### Markov state modelling

Each seeded MD trajectory was reduced to the following features: the RR–hinge–CR angle, the hinge RMSD to the inactive conformation, and the PBC RMSD to the inactive conformation, using cpptraj. Residue masks are outlined in ESI Table 3.† All Markov state modelling here was performed using pyemma version 2.5.7.,^46^ with a lag time, *τ*, of 25 ns for all MSMs based on implied timescales (ITS) shown in ESI Fig. 8.† The data from *apo* EPAC1, EPAC1–cAMP, EPAC1 L273W, EPAC1–I942 was pooled together and clustered into 300 microstates using *k*-means clustering. The equilibrium probability of each microstate was computed from the resulting MSMs. The microstates were assigned to 3 metastable states: active, inactive, and intermediate. The state with the lowest domain angle, hinge and PBC RMSD values was considered the inactive state, while the state with the highest values of these features was considered active. The intermediate state is characterised by a low hinge RMSD value (“inactive”-like) and a high PBC RMSD value (“active”-like), as outlined in Shao *et al.*^25^ Originally, PCCA was applied, however this algorithm did not yield a set of states with well-defined state clusters that satisfied the conditions above (ESI Fig. 4†). Therefore, the macrostate assignment was done manually. The metastable state centres were assigned based on feature values observed during equilibrium and seeded MD simulations (see Section and ESI Table 4†). The total macrostate probabilities were computed by summing over the probabilities of the assigned microstates.

To assess the robustness of the models, the state probabilities of each system were bootstrapped by resampling. For each system, the seeded MD trajectory pool was resampled with replacement to select 200 trajectories. With this new resampled pool of trajectories, a new MSM was built, using the same micro- and macro-state assignments. The probabilities of inactive, intermediate, and active states were computed as above. This was repeated for 100 iterations, yielding a distribution of probabilities for each system. The mean values were used to report metastable populations, and the standard deviation of each distribution was used to report statistical uncertainties. Models where the conformational space is well sampled will show a smaller statistical uncertainty, as excluding a few trajectories should not significantly change the final probability values. The median and inter-quartile range (IQR) between quartiles 1 and 3 is reported to indicate the spread of the bootstrapped probabilities.

Conformational ensembles were generated by drawing 10 000 frames from the seeded MD pool. The probability of any frame to be sampled was based on the MSM-computed equilibrium probability of the microstate that frame was assigned to. The distances and dihedrals discussed in the results below were computed from these ensembles using cpptraj. In the case of EPAC1_L273W_–cAMP simulations, the active state ensemble was also recreated similarly, using only microstates assigned to the active state. This was necessary as the active state is barely sampled in the full equilibrium ensemble of this system.

## Results and discussion

### Equilibrium and steered MD simulations define the metastable states of EPAC1

Equilibrium MD simulations in both active and inactive conformations were carried out for a duration of 1 μs each. The following features were computed: RR–hinge–CR inter-domain angle, hinge RMSD to the inactive conformation, and PBC RMSD to the inactive conformation (ESI Fig. 5a† and 2). The domain angle for the inactive conformation remained below 25°, and in the region of 125–150° for the active conformation. The hinge RMSD values were similarly well separated and stable, however PBC RMSD showed some overlap between the active and inactive conformations. This occurs because the change in PBC conformation is very small between active and inactive EPAC1 states. These features were used to subsequently build MSMs.

These results were sufficient to describe the active and inactive states of EPAC1, by using feature values observed during the equilibrium MD simulations. As the intermediate conformation is defined as a mix of hinge “out” (or inactive-like) and PBC “in” (or active-like) conformations, the definition of the hinge and PBC coordinates was also straightforward. However, the appropriate domain angle values to be used for the definition of the intermediate state were not evident from the equilibrium MD simulations, as EPAC1 remained in its active or inactive starting conformation on a 1 μs timescale. To choose a suitable domain angle value for the intermediate state, a simulation steering EPAC1 from inactive to active conformation (outlined below) was aligned to an EPAC2 structure with RAP bound (PDB ID: 3CF6). Only the catalytic regions were used for alignment, to capture the translation of the regulatory region. Pairwise C(α)–C(α) distances between all EPAC1 RR and all RAP residues were computed for the sMD simulation, taking the smallest one as the EPAC1(RR)–RAP distance. This distance was plotted against the domain angle, to provide a direct relation between the inter-domain angle and the occlusion of the RAP binding site by the RR (ESI Fig. 5b). While the domain angle increased from 20° to 60°, the minimum Cα–Cα distance remained low, but jumped up as the domain angle increased from 60° to 95° (ESI Fig. 5B†). Thus, the inter-domain angle of 60° was used to define the centre of the intermediate state, and values of 30° and 100° for the centres of the inactive and active states respectively.

### Markov state modelling captures activation by cAMP

**Fig. 3:**
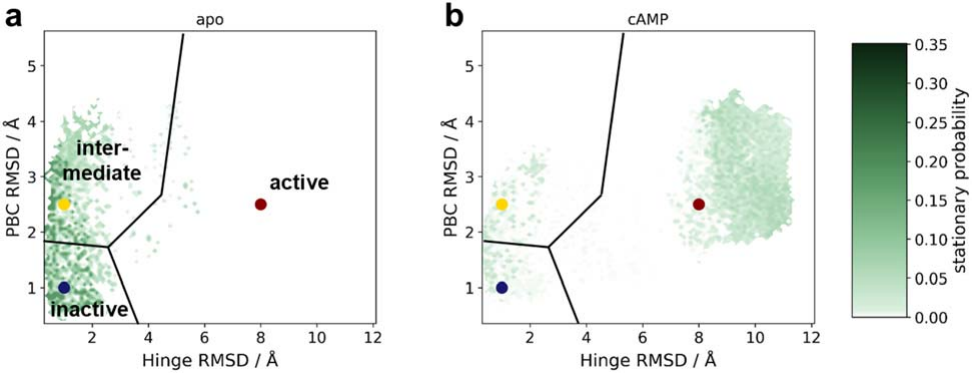
The equilibrium probability density map about the hinge and PBC RMSD to inactive conformation space for (a) *apo* EPAC1 and (b) EPAC1–cAMP. On top, the macrostate centres and the resulting space partitioning are depicted: inactive (blue), intermediate (yellow) and active (red). Equivalent plots for the remaining five systems can be found in ESI Fig. 9.†

The sMD/MSM workflow shown in Fig. 2a was applied to *apo* EPAC1 and the EPAC1–cAMP complex. Example sMD results are shown in ESI Fig. 6.† The probability density of each state is shown in Fig. 3 for the apo and cAMP bound systems, along with the macrostate partitioning is plotted as a function of the hinge and PBC RMSD features.

**Fig. 4:**
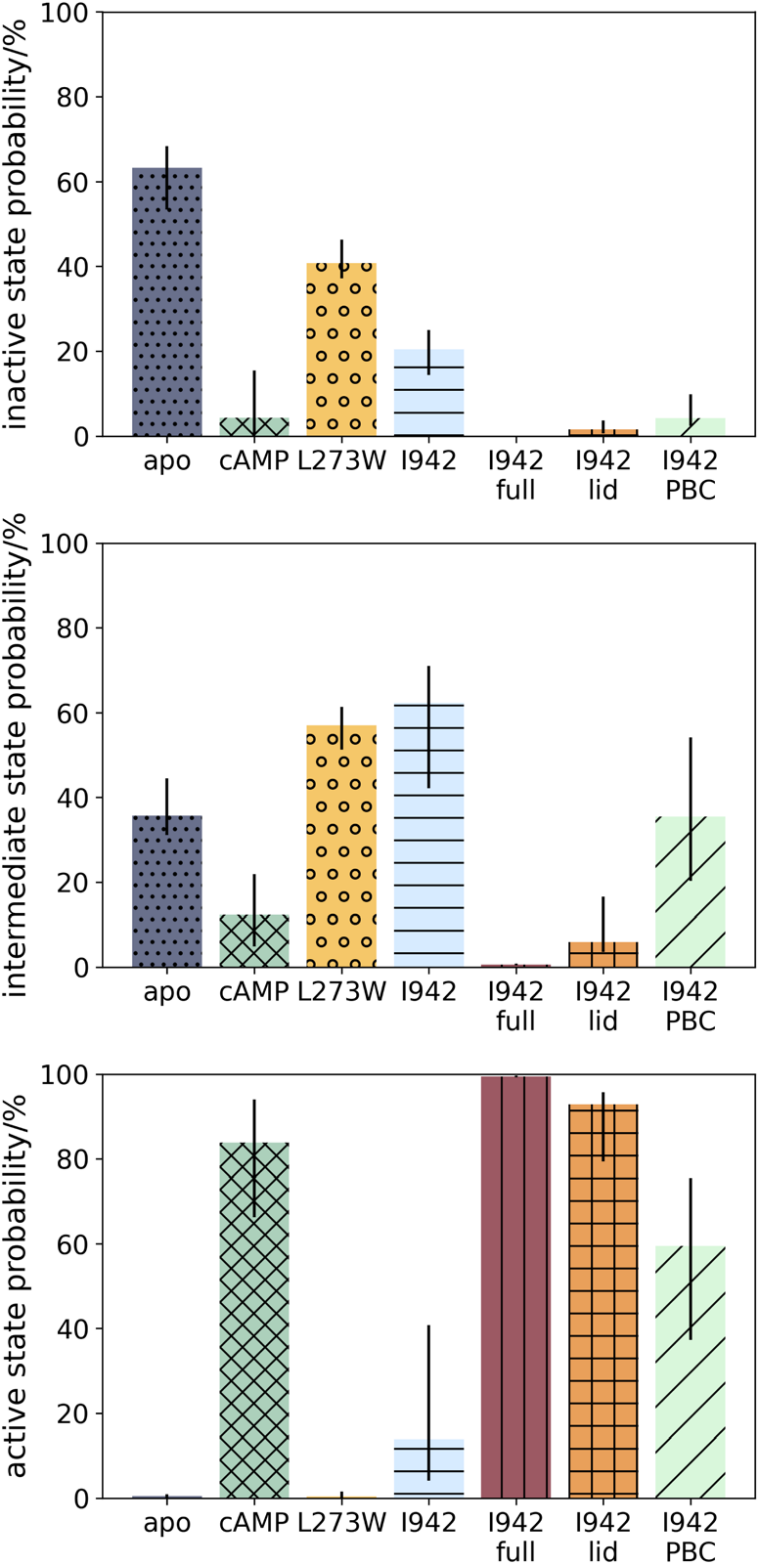
The bootstrapped probabilities of the inactive, intermediate and active EPAC1 states for each system described in this work. The bar values are the median values of the bootstrapped probability distributions, while the black vertical bars show the inter-quartile range between quartiles 1 and 3.

In agreement with experimental results, *apo* EPAC1 is modelled as mainly adopting the inactive conformation (63%, Inter Quartile Range (IQR) 53–68%), some intermediate conformation (36%, IQR 31–44%), and no active conformation (0%, IQR 0–0%) (Fig. 4). These values are reflected in the stationary probability distribution shown in Fig. 3, where most of the density is seen at low hinge and PBC RMSD values, and no density is seen in the high value region. Shifting the coordinates of the intermediate centre to adjust the states partitioning between the three macro-states only had a moderate effect on the macrostate populations and did not lead to qualitatively different interpretations of the population shifts (ESI Fig. 7†). In the presence of cAMP, the probability of an active state increases to 84% (IQR 66–94%), demonstrating that the sMD/MSM approach applied here has captured strong activation by cAMP. Furthermore, the stationary probability density of cAMP in Fig. 3 shows an increase in the probability density in the high regions of the hinge and the RMSD values of PBC, corresponding to the active state.

**Fig. 5:**
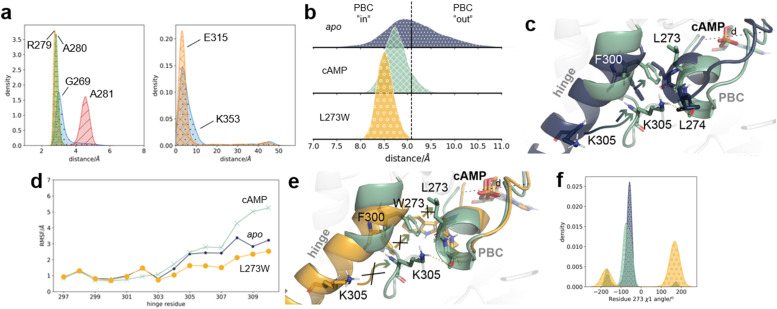
Comparison of conformational ensembles in *apo* EPAC1, EPAC1–cAMP, and EPAC1_L273W_–cAMP. (a) The distribution of hydrogen bond distances between EPAC1 residues and cAMP depicted in Fig. 1c. Left: the PBC region, G269 (blue dots), R279 (orange crosses), A280 (green horizontal lines), and A281 (red diagonal lines). Right: lid region, K353 (blue dots) and E315 (orange crosses). In the lid region, the large distances correspond to the inactive/intermediate conformations when the lid region is positioned away from cAMP. (b) The backbone atom distance between G269 and residues 279–281 in *apo* EPAC1 (blue dots), EPAC1–cAMP (green crosses), and EPAC1_L273W_–cAMP (yellow circles). The median distribution value of *apo* EPAC1 is shown in a black dashed line. (c) The conformations of the hinge and the PBC in *apo* EPAC1 (dark blue) and when cAMP is present (green). Residue rearrangements are indicated by arrows. (d) The RMSF of each of the hinge residues to the inactive conformation for *apo* EPAC1 (small dots, dark blue), EPAC1–cAMP (crosses, green), and EPAC1_L273W_–cAMP (large dots, orange). (e) The hinge and PBC conformation observed in WT EPAC1–cAMP (green) and EPAC1_L273W_–cAMP (yellow). (f) The residue 273 *χ*1 angle in *apo* EPAC1 (dark blue dots), with cAMP present (green crosses) and the L273W mutant (yellow circles).

To further analyse the conformational changes induced by cAMP, 10 000 frames were sampled from the seeded MD data for *apo* EPAC1, as well as EPAC1–cAMP, according to the MSM state probabilities. Analysis of the resulting conformational ensemble confirms that cAMP maintains hydrogen bond interactions with the phosphate binding cassette (PBC) throughout seeded MD simulations, as shown in Fig. 5a. The distance between these two ends of the PBC, shown in Fig. 5c as distance *d*, can be used to monitor the “in”/“out” conformation of the PBC. *Apo* EPAC1 shows a large fluctuation between the two conformations, with the distance varying between 8 Å and 11 Å (Fig. 5b). This agrees with the high population of the intermediate state seen in *apo* EPAC1 (Fig. 3 and 4). When cAMP is present, the distance distribution is much narrower with a median value of 8.5 Å, as the PBC is pulled into the “in” conformation (Fig. 5b and c).

While the PBC rearrangement is a relatively small structural change, this shift is sufficient to open up the space between the hinge and the PBC, moving L273 out and up, and allowing F300 of the hinge to take its place. The hinge “melts” at the C-terminus, swinging the lid region towards the cAMP binding site. The change in the conformation of the hinge C-terminus is shown through the root-mean-square-fluctuations (RMSF) of each residue in Fig. 5d. Residues 303–309 show increasingly higher fluctuations in the cAMP system. ESI Fig. 10† shows the distance between D750 and Q168, one residue pair making up the ionic latch interactions outlined in ESI Fig. 1,† which lock the catalytic and regulatory regions together near the RAP binding site in the inactive conformation. There is a large increase in this distance observed when cAMP is present, where a peak at 40–60 Å corresponds to the active conformation. The higher energy conformation of the disordered hinge is stabilized by K305 reaching across and forming a hydrogen bond to the L274 of the PBC (Fig. 5c and ESI Fig. 11†). Additionally, when EPAC1 is activated, K353 and 315 residues of the “lid” region^11^ are positioned near cAMP, creating a ligand-mediated hydrogen bond network between the catalytic and regulatory regions (Fig. 1c and 5a). Therefore, cAMP appears to act in a dual fashion, both inducing the activation of EPAC1 and then stabilizing the active state, once it is reached. The importance of each action is investigated below through analysis of the L273W mutant and I942 simulations.

### The L273W mutant populates an intermediate state

The L273W mutant was shown to prevent activation of EPAC1, even in presence of cAMP. It has been proposed that this mutation disrupts the packing interaction with residue F300 and stabilizes the helical structure of the hinge.^12^ sMD/MSM calculations were also applied to the L273W EPAC1 in complex with cAMP to further validate the protocol. The calculations revealed that the hinge does not melt at its C-terminus, and the L273W mutant shows a 0% (IQR 0–1%) active state probability, even though cAMP is present (Fig. 4). The inactive and intermediate states are similarly populated (41%, IQR 37–46%, and 57%, IQR 51–61% respectively). These findings are consistent with the NMR measurements of Shao *et al.*^25^

Comparison of the PBC conformation in the MSM probability-weighted conformational ensemble shows that the PBC is slightly further “in” than in the WT cAMP complex (Fig. 5b). However the mutant W273 does not adopt the same conformation as L273 in the presence of cAMP, indicated by the *χ*1 angle shown in Fig. 5f. The larger tryptophan side-chain is too sterically hindered to move upwards, and instead remains in the space between the hinge and the PBC, shown in Fig. 5e. This allows the tryptophan to π–stack with F300, which in turn further stabilizes the helical conformation of the hinge. This causes the EPAC1_L273W_–cAMP complex to favour the intermediate state. cAMP still acts on the PBC, pulling it into the “in” conformation (as well as the large W273 pushing it out), but the change in interaction between W273 and F300 means that the “out” hinge conformation is even further stabilized. In Fig. 5d, the L273W hinge fluctuations are lower than even in *apo* EPAC1. This increased stabilization of the hinge also allows for stronger ionic latch interactions at the RR/CR interface (ESI Fig. 10). The “inactive” microstates highly populated by this system are observed at higher PBC RMSD values (ESI Fig. 9†) because the larger W273 residue side chain separates further the PBC and hinge regions.

In the EPAC1_L273W_–cAMP complex, cAMP maintains the same protein–ligand interactions as observed in the WT EPAC1–cAMP system (ESI Fig. 12†). The hydrogen bonds to the “lid” region are not present in the full MSM re-weighted ensemble, as the active state is required to position the residues in place for the hydrogen bond interactions. To ensure that, when the system was in the active state, these stabilizing interactions were possible, only the active state sub-ensemble was recreated using the MSM probabilities. The active state sub-ensemble of EPAC1_L273W_ shows that the stabilizing hydrogen bonds are indeed prevalent in these conformations (ESI Fig. 12†). This confirms that the L273W mutation does not change the cAMP binding pose, but instead changes the hinge–PBC interactions, preventing activation by stabilizing the inactive hinge conformation.

### I942 does not induce the same conformational changes than cAMP in the PBC and hinge regions

I942 was shown to be a partial activator of EPAC1,^23^ and proposed to stabilise an intermediate conformation, that lies between known active and inactive EPAC1 states.^25^ Application of our sMD/MSM protocol shows that EPAC1 in complex with I942 occupies the active state with 5% (IQR 1–23%) probability, and the intermediate state with 56% (IQR 49–59%) probability (Fig. 4). The small increase in active state probability over EPAC1 alone is consistent with the partial activation of EPAC1 by I942 observed experimentally. Likewise there is also an increase in the intermediate state probability compared to the *ca.* 40% observed with *apo* EPAC1. Shao *et al.*'s NMR study suggested that EPAC1–I942 populates the active state with a *ca.* 30% probability. The lower values observed here may be due to inaccuracies in the molecular model or a different partitioning of EPAC1's conformational space into active/intermediate/inactive states between MD and NMR methodologies.

**Fig. 6:**
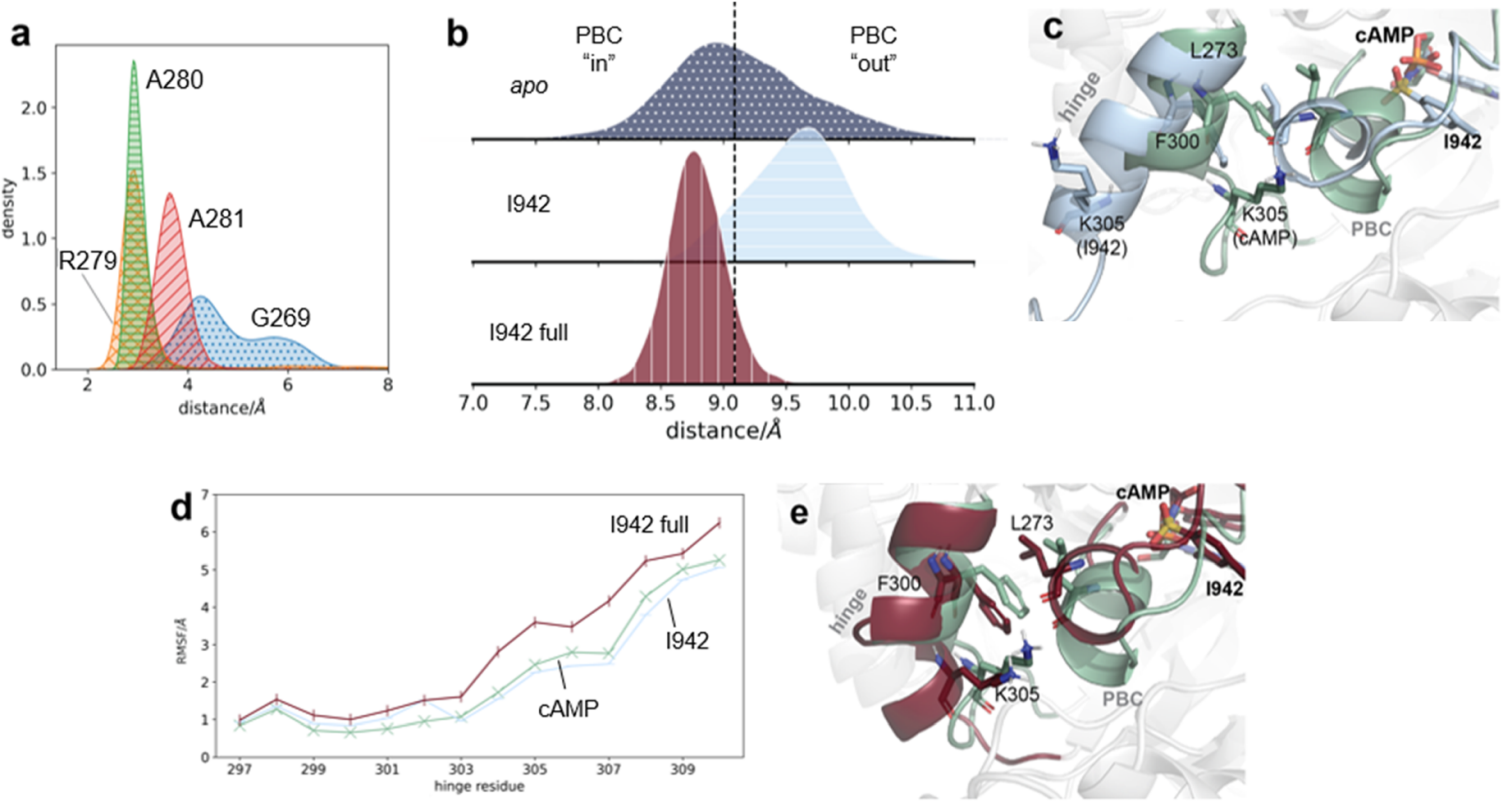
Interactions of unrestrained and restrained I942 with EPAC1. (a) The hydrogen bond distances between unrestrained I942 and G269 (blue dots), R279 (orange crosses), A280 (green horizontal lines), and A281 (red diagonal lines). (b) The backbone atom distance between G269 and residues 279–281 in *apo* EPAC1 (blue dots), EPAC1–I942 (blue horizontal lines), and EPAC1–I942 with I942 restrained to the lid region and PBC (red vertical lines). The median distribution value of *apo* EPAC1 is shown in a black dashed line. (c) The conformations of the hinge and the PBC in EPAC1–cAMP (green) and EPAC1–I942 (light blue) conformational ensembles. (d) The RMSF values of hinge residues, for EPAC1–cAMP (green, crosses), EPAC1–I942 (light blue, horizontal lines, and EPAC1–I942 restrained (red, vertical lines). (e) Representative conformations of the hinge and the PBC in conformational ensembles of EPAC1–cAMP (green) and EPAC1–I942, when I942 is restrained to the lid region and the PBC (red).

The partial activation of EPAC1 by I942 in the sMD/MSM calculations is also reflected in the distances observed between ionic latch residues of the catalytic and regulatory regions. Hydrogen bonding is interrupted, but the distance is only slightly larger than observed in *apo* EPAC1, and does not reach the 40–60 Å range observed with cAMP (ESI Fig. 10†). While I942 forms hydrogen bond interactions with residues 279–281, similar to cAMP, the interaction with G269 is not always observed (Fig. 6a). Therefore the distance between the two ends of the PBC remains higher than observed with cAMP (Fig. 6b), corresponding to a more “out” PBC conformation. Fig. 6c compares the conformations of the PBC and the hinge with cAMP and I942, and illustrates how the gap required to accommodate the disordered hinge C-terminus between the hinge and the PBC is not present, preventing full activation of EPAC1. This is also reflected in the RMSF values of hinge residues 303–309 in EPAC1–I942, which show an increase in fluctuation but do not reach the same levels as cAMP (Fig. 6d). Additionally, the naphthyl group on I942 cannot form the same active state stabilizing interactions than cAMP with K353 and E315 from the lid region. Therefore I942 fails both to fully activate EPAC1, and to stabilize the active state once it is populated.

To investigate the importance of the protein–ligand interaction differences between I942 and cAMP outlined above, I942 was remodelled with weak flat-bottom distance restraints applied between I942–K353 (lid region) and I942–G269 (PBC), to mimic the hydrogen bonds seen with cAMP (ESI Fig. 3†). Although the restraints to the lid region directly stabilise the active state, they had little effect on the sMD simulations because the steering force was much stronger than the distance restraints forces. All restraints parameters are shown in ESI Table 2.† With these restraints in place and following the same protocol as above, the modelled active state probability of EPAC1–I942_restrained_ jumped to 99% (IQR 99–100%) (Fig. 4b). The complete displacement of the RR is also evident in the IL distance, where only distances above 20 Å are observed (ESI Fig. 10†). The I942–G269 restraint was sufficient to decrease the distance between the N- and C-termini of the PBC, bringing it into the “in” conformation (Fig. 6b). The increased space between the hinge and the PBC also allowed for greater disordering of the hinge, as reflected in the higher hinge RMSF values (Fig. 6d). The active state probability of the EPAC1–I942_restrained_ system is higher than that of the EPAC1–cAMP system because of the use of artificial distance restraints to attract I942 to residues G269 and K353. While those distance restraints were chosen to mimic the directional hydrogen-bonding interactions seen in the EPAC1–cAMP simulations, their parameters do no match exactly the energetics of the EPAC1–cAMP hydrogen bonding interactions between those two residues.

**Fig. 7:**
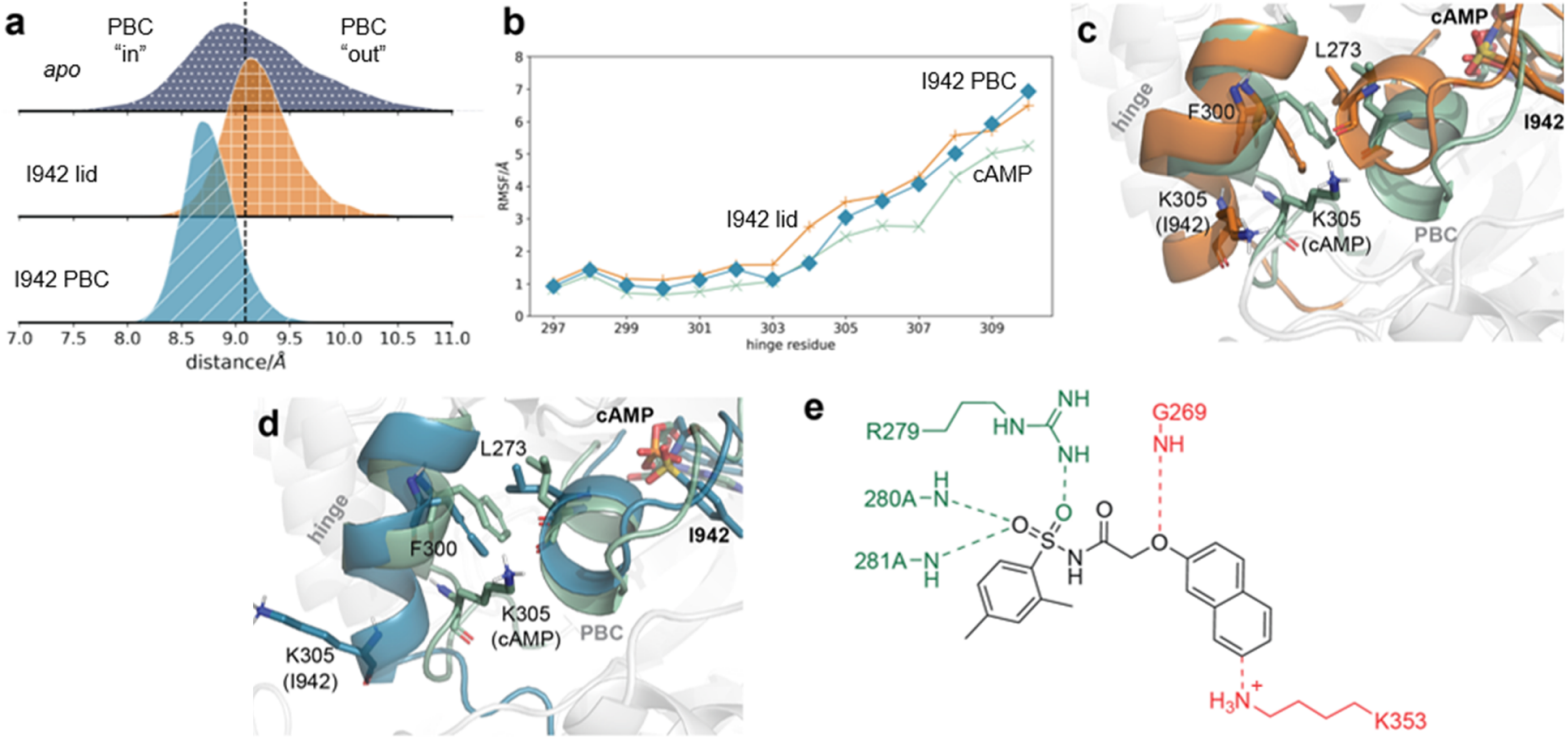
Interactions of I942, restrained only to the lid region or the PBC, with EPAC1. (a) The backbone atom distance between G269 and residues 279–281 in *apo* EPAC1 (blue dots), EPAC1–I942 restrained to the lid (orange squares), and EPAC1–I942 restrained to the PBC (teal diagonal lines). The median distribution value of *apo* EPAC1 is shown in a black dashed line. (b) The RMSF values of hinge residues, for EPAC1–cAMP (green, crosses), EPAC1–I942 restrained to the lid (orange, plus), and EPAC1–I942 restrained to the PBC (teal, diamonds). (c) Representative conformations of the hinge and the PBC in conformational ensembles of EPAC1–cAMP (green) and EPAC1–I942, when I942 is restrained to the lid region only (orange). (d) Representative conformations of the hinge and the PBC in conformational ensembles of EPAC1–cAMP (green) and EPAC1–I942, when I942 is restrained to the PBC only (teal). (e) Proposed model for development of improved I942 derivatives. Interactions to maintain are shown in green (residues 279–281), and new interactions to target are shown in red (residues 269 and 353).

Insights into the relative effects of the lid and PBC distance restraints were sought by repeating the calculations with only one set of distance restraints applied at a time. When only the lid region restraints were applied, the active state probability of EPAC1 was only slightly decreased to 94% (IQR 88–97%) (Fig. 4). The ionic latch distances are also very similar to when both sets of restraints are applied (ESI Fig. 10). As the PBC distance restraints were not applied in this case, the PBC still populates more of the “out” conformation (Fig. 7a). However, compared to fully unrestrained simulations, the PBC N- to C-terminus distance has decreased. Thus lid stabilisation of the active state facilitates rearrangement of the PBC region.

When the simulations were repeated with PBC distance restraints only the active state probability decreased to 52% (IQR 31–67%), which is below the active state probability seen for the EPAC1–cAMP system. As expected, the PBC region mainly adopts the “in” conformation, indicating that tightening interactions between I942 and G269 is indeed sufficient to fully shift the PBC conformation from “out” to “in”. This PBC rearrangement is sufficient to partially disrupt the ionic latch (ESI Fig. 10). The hinge region RMSF values are similar when only the PBC or the lid restraints are applied. This shows that the PBC shift from “out” to “in” is also sufficient to allow melting of the hinge. However, the lower active state probability observed when only PBC restraints are applied suggests that additional lid–ligand interactions are necessary to stabilise the active state conformation.

Given the above findings, we propose that the derivatives of I942 with enhanced EPAC1 activation should be pursued by replacement of the naphthyl group with moieties designed to engage in hydrogen bonding interactions with K353 (Fig. 7e). Ligand modifications to engage G269 in addition to residues 279–281 should also allow for greater PBC rearrangement upon ligand binding and greater stabilisation of EPAC1's active state.

## Conclusions

This work has produced a series of molecular models of the protein EPAC1 in three distinct functional states (inactive, intermediate, and active). We note that the partitioning of conformational space between inactive and intermediate states is somewhat arbitrary. The distance between G269 and residues 279–281 was used here to describe the changes in PBC conformation. Other descriptors, such as change in the activator pocket volume could have been used. It is therefore advisable to assess detailed probability distribution plots built from selected features such as in Fig. 3 before further simplifying protein dynamics to two or three macrostates. However, the exact geometric boundaries between the intermediate state and active/inactive states is less significant for developing full activators since the active/inactive state partitioning is much clearer.

Caveat aside, Markov State Modelling correctly captured the activation of EPAC1 by cAMP, as well as the interruption of said activation by the L273W mutation (Fig. 4). The MSMs allow quantification of the effects of ligands on protein conformational preferences, and output equilibrium conformational ensembles that can be interrogated to support design hypotheses. The dynamics of these ensembles confirm the previously reported dual action of cAMP, that is the change in PBC conformation and stabilisation of active state *via* the lid region.^11^ Comparison of the WT EPAC1 and EPAC1_L273W_ ensembles also confirms that the mutation decouples the PBC from the hinge, but does not interfere with cAMP binding.^12^ The mutated W273 residue does not shift out of the space between the hinge and the PBC to allow for stabilisation of a disordered hinge C-terminus, and also actively stabilises the helical conformation of the hinge through π–stacking interactions with F300 (Fig. 5d, f, and g). Insights into the contribution of these interactions to the allosteric mechanism could be further sought *via* additional computational or experimental studies of double mutants, for instance EPAC1_L273W/F300L_–cAMP.

Our modelling also supports findings by Shao *et al.*^25^ on the partial activation of EPAC1 by I942. The presence of I942 increases the probabilities of both the active and intermediate states, compared to the *apo* EPAC1 model. In comparison with EPAC1–cAMP, only a slight movement towards an “in” conformation of the hinge is observed, and the PBC shift to the “in” conformation is incomplete. Since our EPAC1 models included the lid present in the catalytic region we could additionally investigate the effect of enforcing protein–ligand distance restraints between I942 and that region. The restraints to the lid region proved extremely effective in stabilizing the active conformation, yielding a model where the active state was almost exclusively populated (Fig. 4). In this restrained model, no ionic latch interactions were observed, indicating a complete exposure of the RAP binding site (ESI Fig. 10). Such active state stabilising interactions have been observed in an X-ray structure^11^ of EPAC2–cAMP(Sp). The present simulations demonstrate that similar interactions occur with EPAC1. Since the EPAC1 construct used in the NMR work of Shao *et al.* did not include the lid region,^25^ this role of the catalytic region in EPAC1 activation could not be evaluated. Consequently, efforts to develop I942 derivatives into full EPAC1 agonists have so far focused on engagement of the PBC.^47^ Herein, use of a full-length EPAC1 model revealed an additional design strategy that features simultaneous engagement of the PBC and lid regions ((Fig. 7e). Having validated the sMD/MSM approach applied here with cAMP, the L273W mutant and I942, further applications of the sMD/MSM methodology can be pursued to guide the design of I942 derivatives. Such work is underway and the characterisation of novel EPAC1 activators will be reported in due course.

Recent work has suggested that anionic membranes may play a role in EPAC1 activation.^48^ The reported synergy of EPAC1 activation by both anionic membranes and cAMP suggests independent mechanisms of allosteric regulation. We posit that the N-terminal disordered region of EPAC1 may be involved in membrane recruitment. Our sMD/MSM methodology could be used in future studies to investigate the role played by this disordered region in anchoring EPAC1 to membranes.

In conclusion, the clinical potential of targeting EPAC1 as a therapeutic strategy is compelling, spanning cardiovascular, metabolic, fibrotic, and oncologic disorders. EPAC1 activation offers significant promise in addressing critical conditions such as heart failure, type 2 diabetes, idiopathic pulmonary fibrosis (IPF), and vascular complications through its ability to modulate intracellular calcium handling, enhance endothelial barrier function, and regulate fibroblast activity. Additionally, its role in promoting brown fat growth and beige adipogenesis introduces a compelling therapeutic avenue for combating obesity by enhancing energy expenditure and reducing metabolic complications. The elucidation of EPAC1 activation mechanisms and the partial agonist behavior of compounds like I942 provide a strong foundation for the rational design of next-generation modulators that can selectively activate EPAC1 while minimizing off-target effects. These findings not only deepen our understanding of EPAC1 dynamics but also pave the way for innovative therapies with the potential to address pressing medical challenges.

## Author contributions

Conceptualization: SY, GB, JM; funding acquisition: SL and JM; investigation: AH, FP, JM; supervision: SL, SY, GB, JM; writing: AH, FP, SL, SY, GB, JM.

## Conflicts of interest

JM is a member of the Scientific Advisory Board of Cresset and the Chemistry Advisory Board of Peptone.

## Supplementary Material

SC-016-D5SC02112J-s001

## Data Availability

Input files and complete workflow to reproduce the protocol are available as examples distributed with the AMMo software at https://github.com/michellab/AMMo. MSM-generated snapshots for each system are available in a Zenodo repository (DOI: 10.5281/zenodo.14901404).
