# Supplementary material for: Elucidation of the mechanism of partial activation of EPAC1 allosteric modulators by Markov state modelling
Source: Chem Sci. 2025 Jul 4;16(32):14771–81. doi: 10.1039/d5sc02112j (PMC12268604; doi:10.1039/d5sc02112j)
Supplement: SC-016-D5SC02112J-s001 [file SC-016-D5SC02112J-s001.pdf]

Supplementary Information (SI) for Chemical Science.
This journal is © The Royal Society of Chemistry 2025

Supplementary Information: Elucidation of the Mechanism of Partial Activation of EPAC1 Allosteric Modulators by Markov State Modelling

Adele Hardie[a,b], Frederick G. Powell[c], Silvia Lovera[d,e], Stephen J. Yarwood[f], Graeme Barker[c], and Julien Michel[a]

[a]EaStCHEM School of Chemistry, University of Edinburgh, David Brewster Road, Edinburgh, EH9 3FJ, United Kingdom
[b]Current address: Sygnature Discovery, Alderley Park, Alderley Edge, Cheshire, SK10 4TG, United Kingdom
[c]Institute of Chemical Sciences, Heriot-Watt University, Riccarton, Edinburgh, EH14 4AS, United Kingdom
[d]UCB, Chemin du Foriest 1, 1420, Braine-l'Alleud, Belgium
[e]Current address: Kesmalea Therapeutics, 8 Bloomsbury Street, London, WC1B 3SR, United Kingdom
[f]Institute of Biological Chemistry, Biophysics and Bioengineering, Heriot-Watt University, Riccarton, Edinburgh, EH14 4AS, United Kingdom

Supplementary Table 1: The collective variable definitions for sMD simulations, as well as the references, target values, and forces at each of the 2 steps of the steering. For the RR RMSD, the target value at step 1 was half of the starting RMSD value. In all cases, backbone atoms were used for steering.

CV	Residues	Reference	Target value /Å	Force /kcal mol^{-1}
**Inactive to Active**				
Regulatory Region	48-339	active<br>active	initial/2, 0	3500, 3500
Hinge	297-310	intermediate<br>active	0, 0	3500, 3500
PBC	270-274	intermediate<br>active	0, 0	3500, 3500
**Active to Inactive**				
Regulatory Region	48-339	inactive<br>inactive	initial/2, 0	3500, 3500
Hinge	297-310	intermediate<br>inactive	0, 0	3500, 3500
PBC	270-274	intermediate<br>inactive	0, 0	3500, 3500

Supplementary Table 2: The flat bottomed restraint parameters used to restrain all ligands. Atoms are indicated using the AMBER atom masks. 2 atoms mean a distance restraint, and 4 atoms mean a torsional angle. r1-4 are the flat bottom well defining points (in Å for distances and degrees for dihedrals), and rk2-3 are the restraint energies (in kcal mol^{-1} $Å^{-2}$ for distances and kcal mol^{-1} deg^{-1} for dihedrals. The ialtd parameter indicates whether energy penalties plateu (ialtd=1) or increase indefinitely (ialtd=0). Restraints with atoms indicated in bold have also been applied in some seeded MD simulations. Note that atom masks are for protein models used in this study, which have an offset of -47 from the full protein.

Atoms	r1	r2	r3	r4	rk2	rk3	ialtd
cAMP							
:233@H :cAMP@O1P	1.3	1.8	3.0	3.50	0.0	150.0	0
cAMP @C4 @N9 @C1' @O4'	46.0	47.0	48.0	49.0	150.0	150.0	0
I942							
:I942@O5' :232@NH1	2.5	3.0	4.0	4.5	0.0	150.0	0
:I942@O :233@N	2.5	3.0	4.0	4.5	0.0	150.0	0
:I942@O :234@N	2.5	3.0	4.0	4.5	0.0	150.0	0
I942 restrained							
:I942@O5' :232@NH1	2.5	3.0	4.0	4.5	0.0	150.0	0
:I942@O :233@N	2.5	3.0	4.0	4.5	0.0	150.0	0
:I942@O :234@N	2.5	3.0	4.0	4.5	0.0	150.0	0
**:I942@O1 :222@N**	2.0	2.5	3.4	3.6	5.0	22.5	1
**:I942@C16 :306@NZ**	2.0	3.5	8.0	9.0	5.0	1.5	1
**:I942@C3 :306@NZ**	6.0	7.0	9.5	10.5	5.0	1.4	1

Supplementary Table 3: AMBER selection masks for the features used to reduce data dimensionality when building MSMs. Note that atom masks are for protein models used in this work, which have an offset of -47 from the full protein.

**Feature**	**type**	**mask**	reference (RMSD only)	alignment mask (RMSD only)
domain angle	angle	:121-125 (@CA,C,N,O) :250-263 (@CA,C,N,O) :701-704 (@CA,C,N,O)	-	-
hinge	RMSD	:250-263 (@CA,C,N,O)	inactive	:122-263 !(@/H)
PBC	RMSD	:223-227 (@CA,C,N,O)	inactive	:122-263 !(@/H)

Supplementary Table 4: The domain angle, hinge RMSD and PBC RMSD values defining the centers to partition microstates into inactive, intermediate, and active metastable states.

Feature	inter-domain angle/°	hinge RMSD/Å	PBC RMSD/Å
Inactive	30	1.0	1.0
Intermediate	60	1.0	2.5
Active	100	8.0	2.5

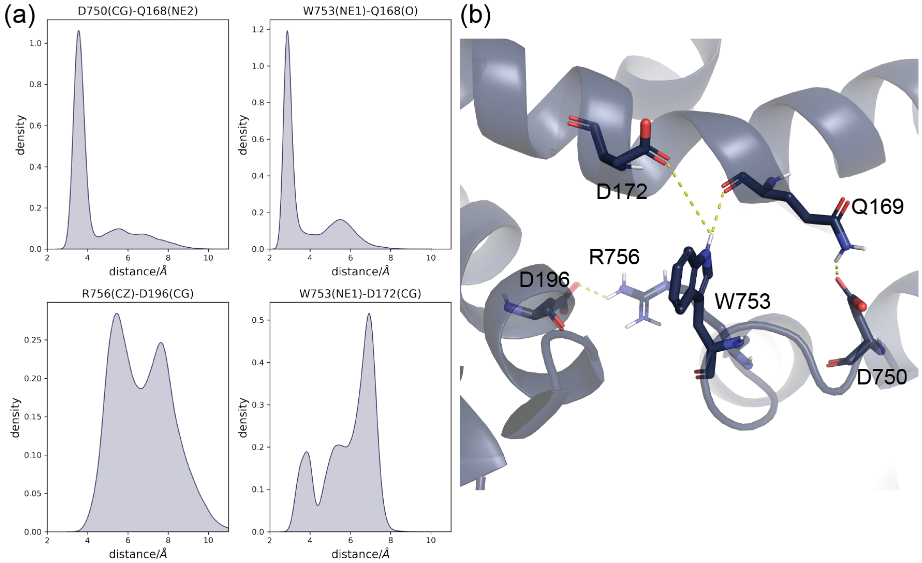

Supplementary Figure 1: Ionic latch of EPAC1. (a) Distances between involved residues during 500 ns equilibrium MD simulation of the *apo* EPAC1 in inactive conformation. (b) Residues involved in the ionic latch.

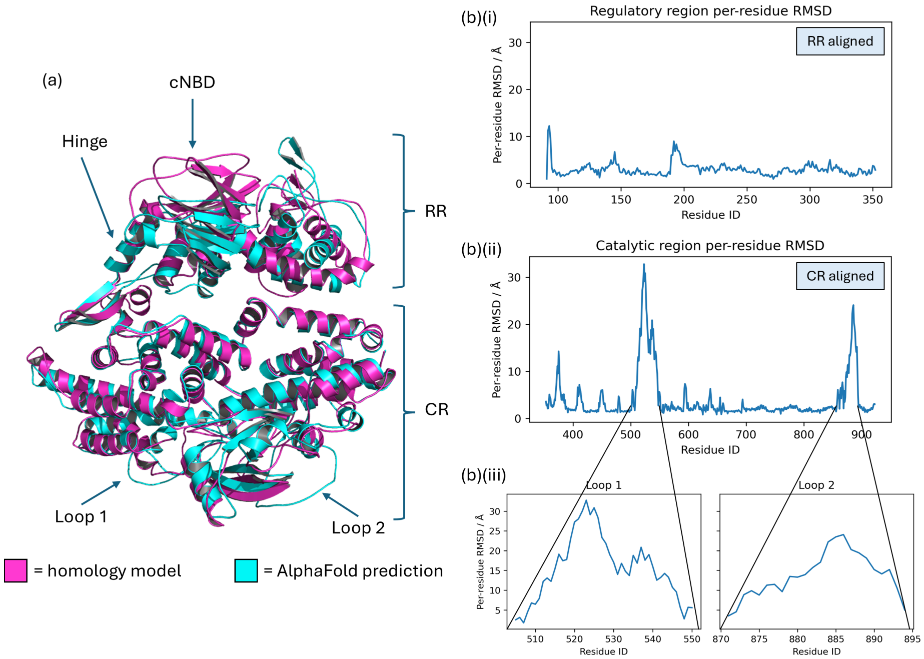

Supplementary Figure 2: (a) The inactive EPAC1 homology model (HM, magenta) and AlphaFold (AF) predicted structure (cyan), following global backbone alignment. Key substructures are labelled. Residues 1-90 have been omitted from the AF structure for clarity. (b) The per-residue RMSD analysis for the HM *vs.* AF EPAC1 structures. The RMSDs for the (i) regulatory and (ii) catalytic regions were measured after aligning the two structures by the corresponding region. This eliminates noise caused by the minor difference in their relative positions. Note that per-residue RMSD analysis starts from residue Q91, as only the AF structure contains residues 1-90. The highly disordered loops within the catalytic region (termed Loops 1 and 2) have been expanded for clarity.

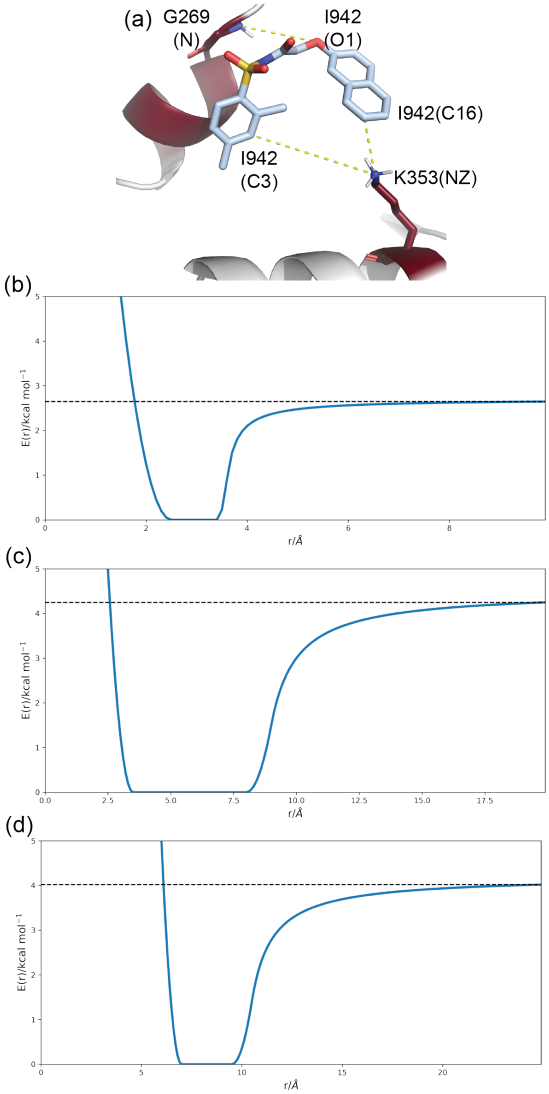

Supplementary Figure 3: Restraints between EPAC1 and I942, mimicking cAMP interactions with the PBC and lid regions. (a) Atoms used for restraints. (b) Restraint energy as a function of distance between I942(O1) and G269(N). The maximum restraint value is shown as a black dashed line. (c) Restraint energy as a function of distance between I942(C16) and K353(NZ). The maximum restraint value is shown as a black dashed line. (d) Restraint energy as a function of distance between I942(C3) and K353(NZ). The maximum restraint value is shown as a black dashed line. This restraint was added to ensure the correct placement of K353, as the distance restraints lack the directionality of hydrogen bonds. All energies were computed as per the AMBER22 manual[**?**].

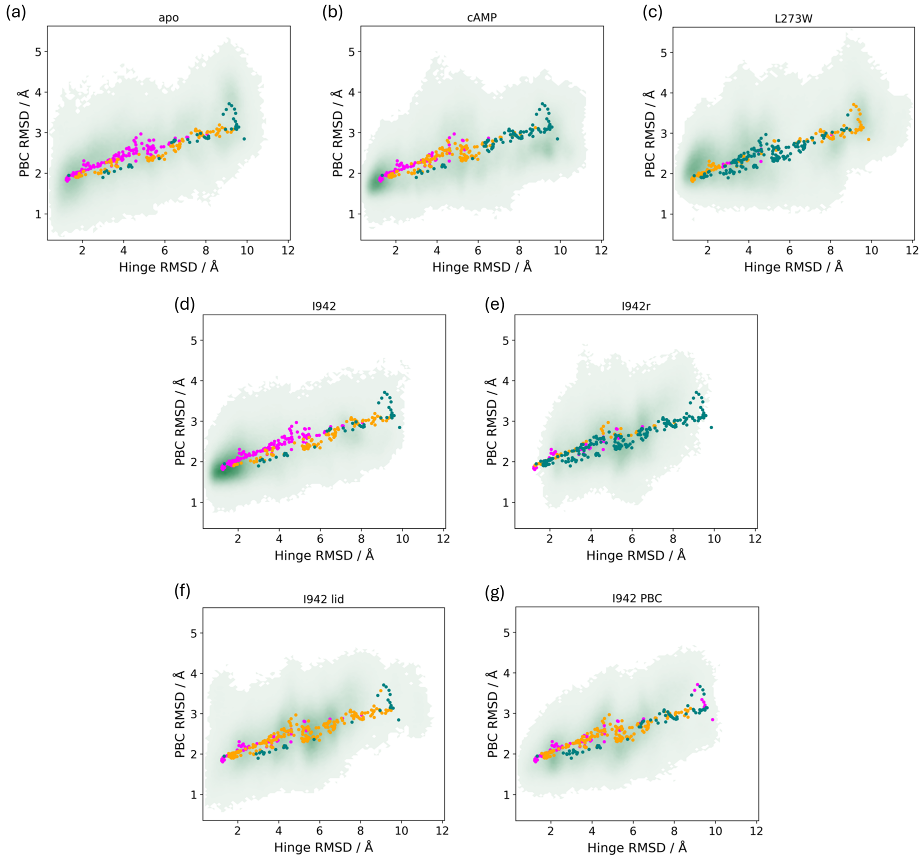

Supplementary Figure 4: The results of PCCA on each of the EPAC1 MSMs used in this study: (a) *apo* (b) EPAC1-cAMP (c) EPAC1$_{\mathrm{L273W}}$-cAMP (d) EPAC1-I942 (e) EPAC1-I942 restrained to both PBC and lid (f) EPAC1-I942 restrained to lid only (g) EPAC1-I942 restrained to PBC only

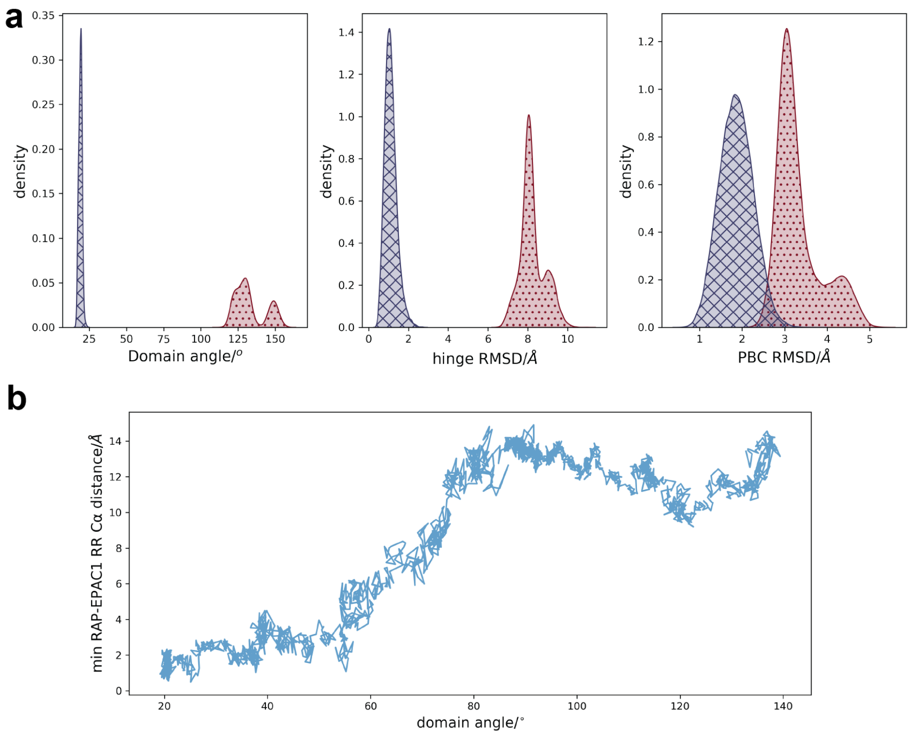

Supplementary Figure 5: Simulations used to define the inactive, intermediate, and active states of EPAC1. (a) The domain angle, (b) hinge RMSD to the inactive conformation, and (c) PBC RMSD to the inactive conformation during 1 μs equilibrium MD simulations of EPAC1 in inactive (blue, crosses) and active (red, dots) conformations. (d) The minimum C(α)-C(α) distances between aligned RAP and EPAC1 regulatory region for each frame of a sMD trajectory, plotted against the associated domain angle values.

(a)

(b)

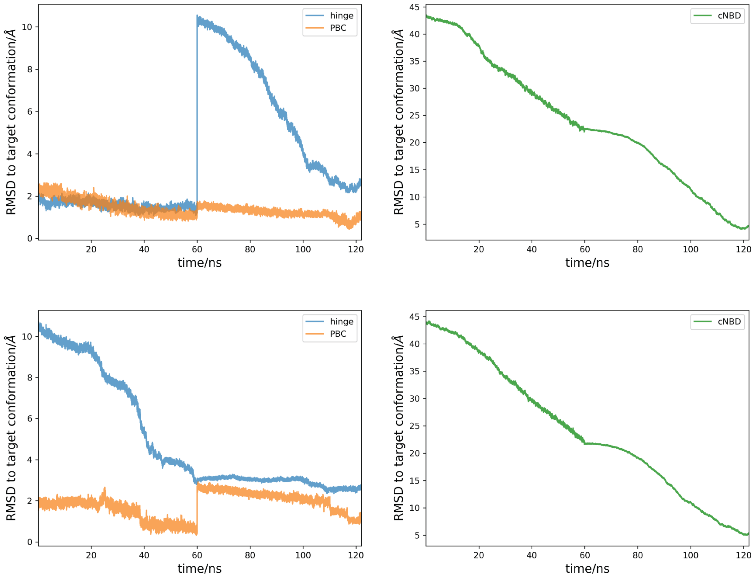

Supplementary Figure 6: Example results of *apo* EPAC1 sMD simulation: (a) inactive to active, and (b) active to inactive. The shift in hinge and PBC RMSD corresponds to changes in RMSD reference conformation.

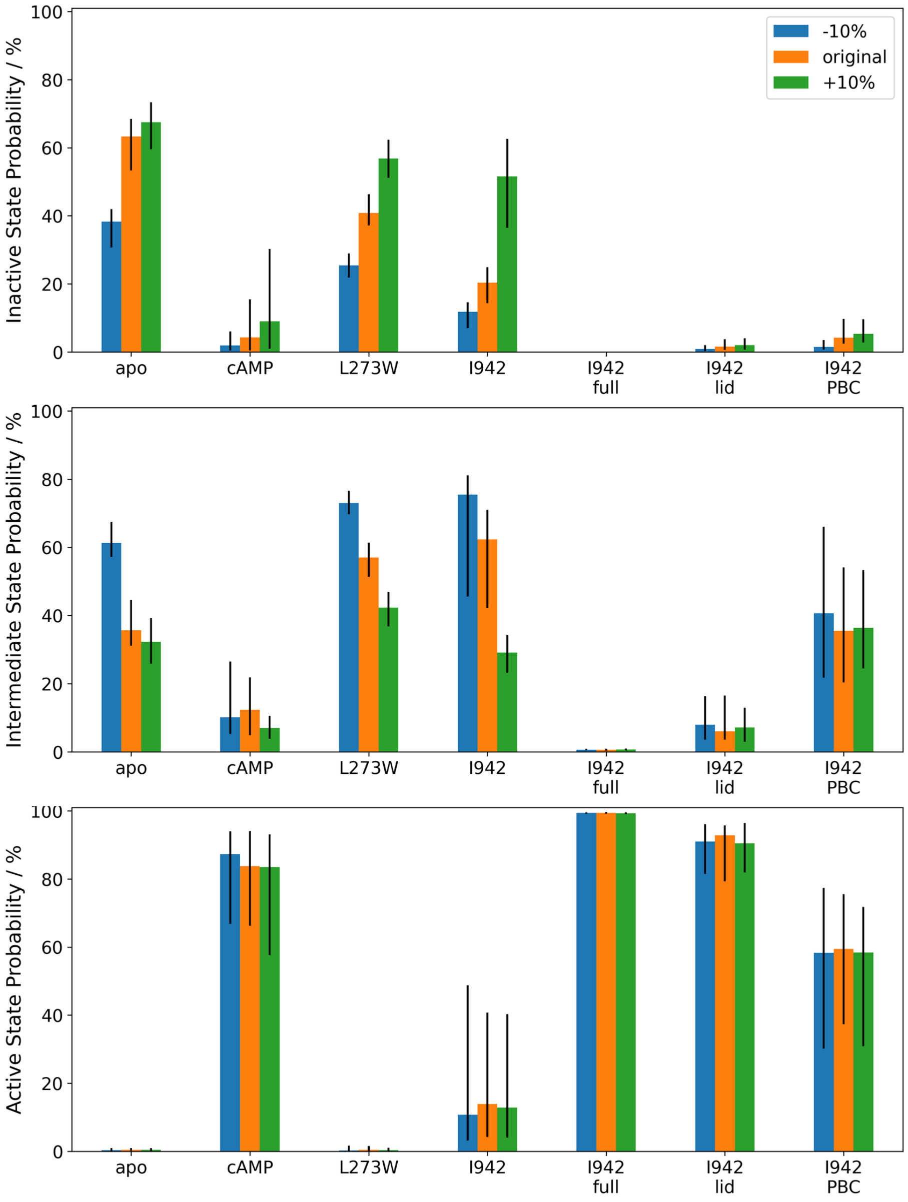

Supplementary Figure 7: The macrostate probabilities for each of our seven systems, highlighting the impact of shifting the intermediate state centre coordinates towards the inactive or active states by 10%. That is modifying the coordinates of the ‘intermediate’ label in Supplementary Table 4 to [54 °, 1 Å, 2.25 Å] and [66 °, 1.1 Å, 2.5 Å] respectively. While the state partitioning affects the absolute state probabilities, none of main conclusions from interpretation of the population shifts are changed.

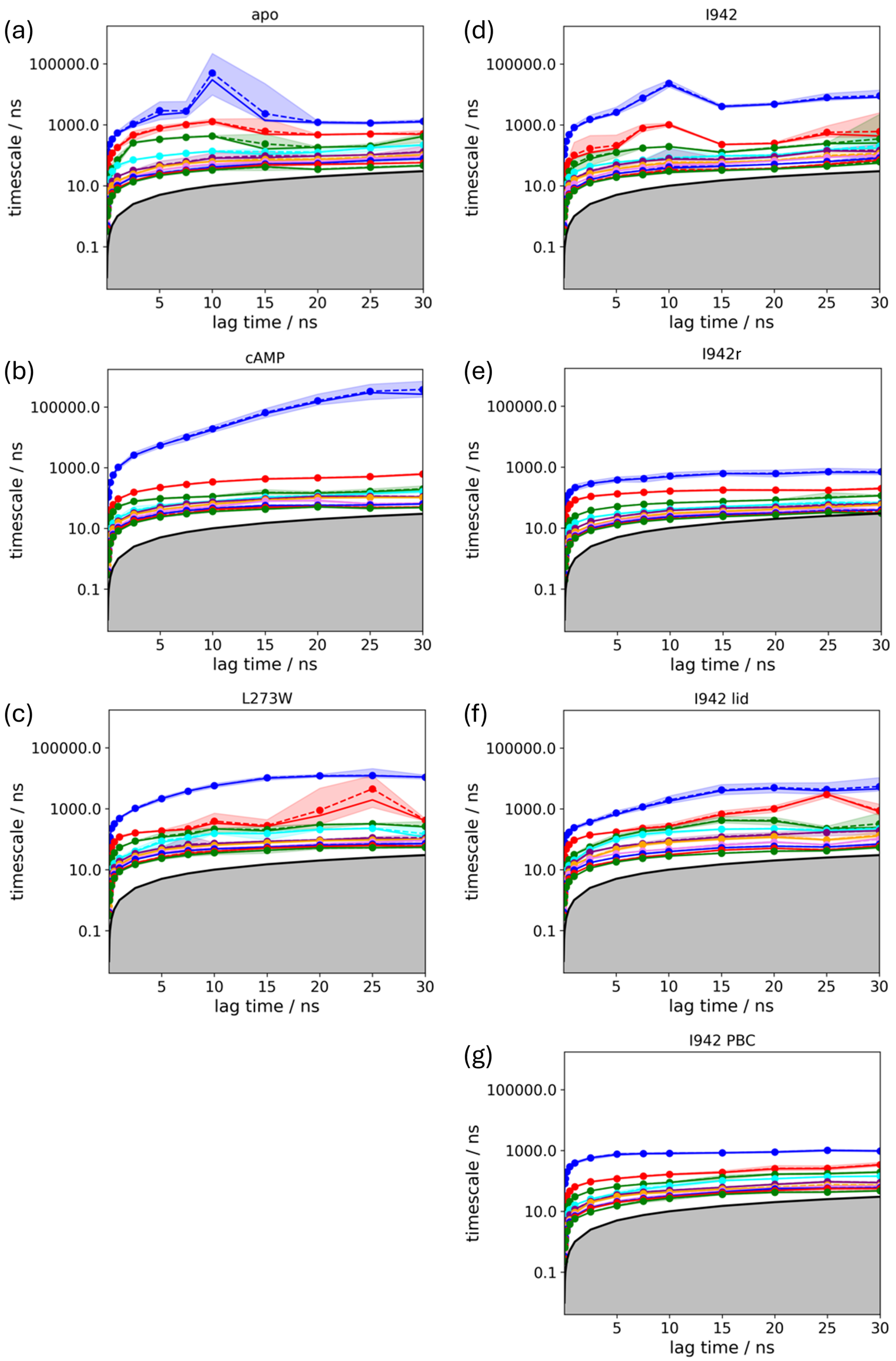

Supplementary Figure 8: ITS plots of the EPAC1 MSMs used in this study: (a) *apo* (b) EPAC1-cAMP (c) $EPAC1_{L273W}$-cAMP (d) EPAC1-I942 (e) EPAC1-I942 restrained to both PBC and lid (f) EPAC1-I942 restrained to lid only (g) EPAC1-I942 restrained to PBC only.

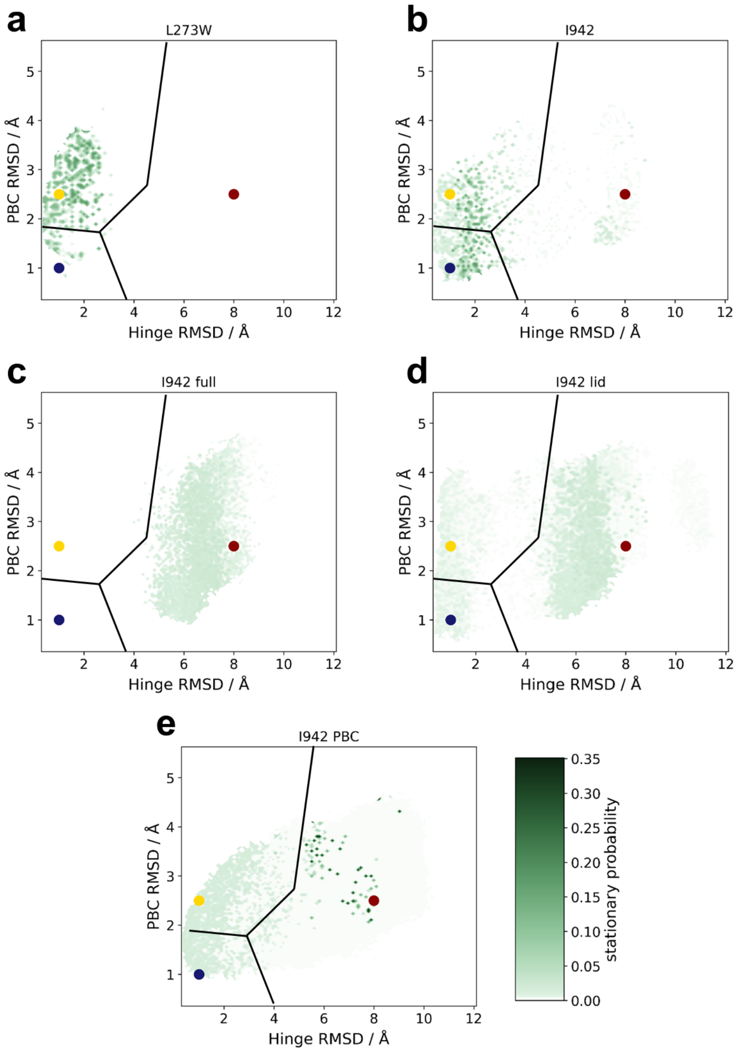

Supplementary Figure 9: The equilibrium probability density map about the hinge and PBC RMSD to inactive conformation space for the remaining systems not shown in Figure 5: (a) L273W, (b) I942 (unrestrained), (c) I942 full, (d) I942 lid, (e) I942 PBC.

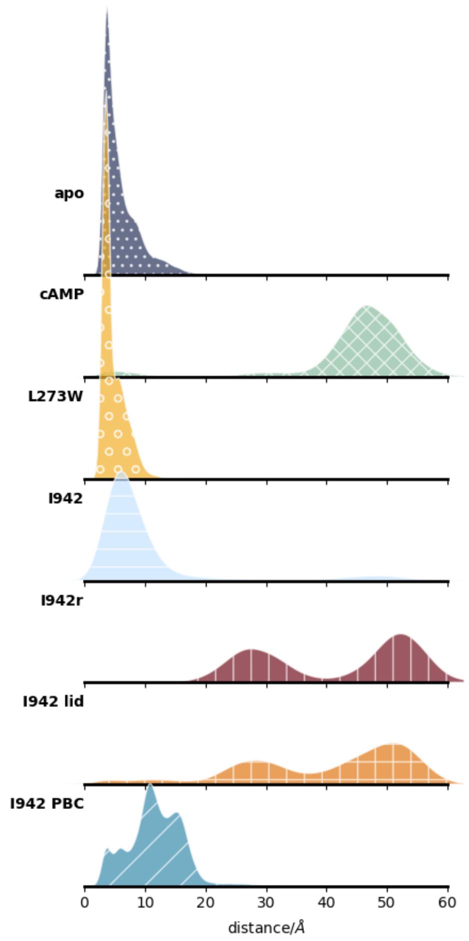

Supplementary Figure 10: The distance between ionic latch residues D750(CG) (catalytic region) and Q168(NE2) (regulatory region), in all systems described in this study.

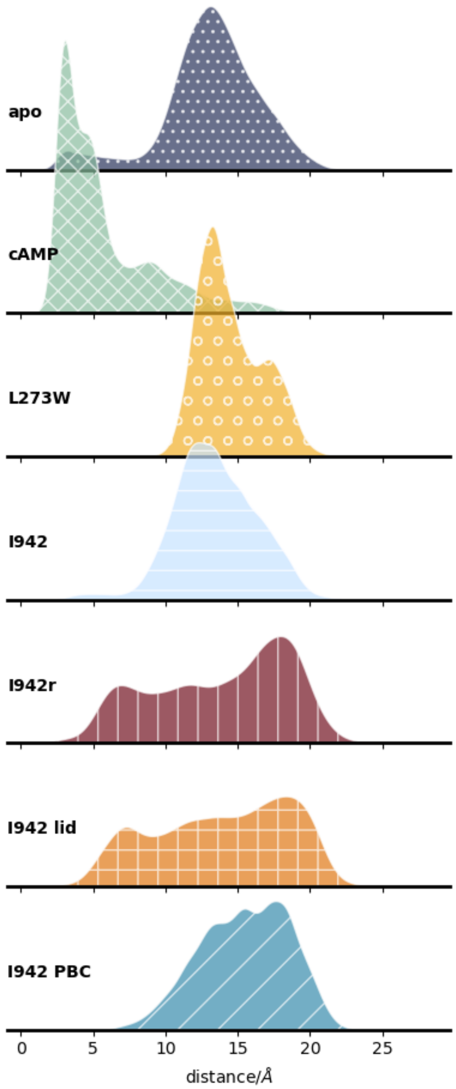

Supplementary Figure 11: The K305(NZ) distance to L274(O), representing the hydrogen bonding between these two residues, for all systems discussed in this study.

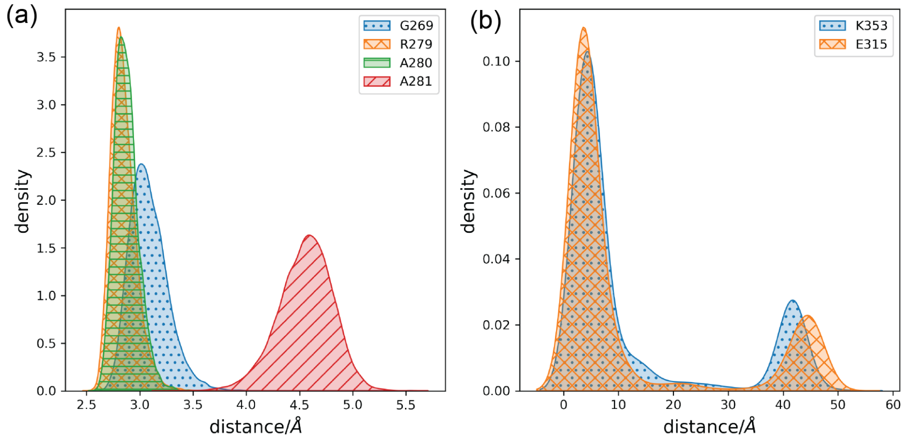

Supplementary Figure 12: The hydrogen bond interactions of cAMP with $\mathrm{EPAC1}_{\mathrm{L273W}}$. (a) hydrogen bonds to the PBC, in the full conformational ensemble of $\mathrm{EPAC1}_{\mathrm{L273W}}$-cAMP. (b) hydrogen bonds to the lid region, in the active state conformational ensemble of $\mathrm{EPAC1}_{\mathrm{L273W}}$-cAMP. As the active state population is very low in the general ensemble, the lid interactions are not apparent there, so only the active state is shown here.
